# The proinsulin/insulin (PI/I) ratio is reduced by postprandial targeting therapy in type 2 diabetes mellitus: a small-scale clinical study

**DOI:** 10.1186/1756-0500-6-453

**Published:** 2013-11-11

**Authors:** Tsuyoshi Ohkura, Kazuoki Inoue, Youhei Fujioka, Risa Nakanishi, Hideki Shiochi, Keisuke Sumi, Naoya Yamamoto, Kazuhiko Matsuzawa, Shoichiro Izawa, Hiroko Ohkura, Masahiko Kato, Kazuhiro Yamamoto, Shin-ichi Taniguchi

**Affiliations:** 1 Division of Cardiovascular Medicine, Endocrinology and Metabolism, Department of Molecular Medicine and Therapeutics, Tottori University Faculty of Medicine, Yonago, Tottori, Japan; 2 Department of Regional Medicine, Tottori University Faculty of Medicine, Yonago, Tottori, Japan

**Keywords:** Proinsulin, Meal tolerance test, Type 2 diabetes mellitus, Glinide

## Abstract

**Background:**

An elevated PI/I ratio is attributable to increased secretory demand on β-cells. However, the effect of postprandial targeting therapy on proinsulin level is unknown. We evaluated the metabolic effect of glinide and sulfonylurea (SU) using the meal tolerance test (MTT).

**Methods:**

MTT was applied to previously untreated Type 2 Diabetes Mellitus (T2DM) subjects. Twenty-two participants were given a test meal (450 kcal). Plasma glucose and insulin were measured at 0 (fasting), 30, 60, 120, and 180 min. Serum proinsulin and C-peptide immunoreactivity (CPR) were measured at 0 and 120 min. Postprandial profile was assessed at baseline and following 3 months treatment with either mitiglinide or glimepiride.

**Results:**

Plasma glucose level at 30, 60, 120, and 180 min was significantly improved by mitiglinide. Whereas, glimepiride showed a significant improve plasma glucose at 0, 180 min. Peak IRI shifted from 120 to 30 min by mitiglinide treatment. The pattern of insulin secretion was not changed by glimepiride treatment. Whereas mitiglinide did not affect the PI/I ratio, glimepiride tended to increase the PI/I ratio. Moreover, although mitiglinide did not affect PI/I ratio as a whole, marked reduction was noted in some patients treated by mitiglinide. PI/I ratio was reduced significantly in the responder group. The responder subgroup exhibited less insulin resistance and higher insulinogenic index at baseline than non-responders. Moreover, the triglyceride level of responders was significantly lower than that of non-responders.

**Conclusions:**

Mitiglinide improved postprandial insulin secretion pattern and thereby suppressed postprandial glucose spike. In T2DM patients with low insulin resistance and low triglyceride, mitiglinide recovered impaired β-cell function from the viewpoint of the PI/I ratio.

**Trial registration:**

UMIN-CTR: UMIN000010467

## Background

Type 2 diabetes mellitus is a heterogeneous disease characterized by insulin resistance and defective insulin secretion [1]. Proinsulin is synthesized and secreted as the precursor form of insulin. Proinsulin level is a predictor of type 2 diabetes mellitus (T2DM), obesity, and cardiovascular disease. The PI/I ratio reflects β- cell dysfunction associated with the onset and progression of T2DM [2,3]. An elevated PI/I ratio is attributable to increased secretory demand on β-cells. One study reported sulfonylurea-treated subjects had a significant elevation in proinsulin/IRI ratio compared with diet-treated subjects, whereas non sulfonylurea hypoglycemic agent-treated subjects (metformin, alpha-glucosidase inhibitor, troglitazone) did not [4]. However, the effect of postprandial targeting therapy on proinsulin level is unknown. It has been reported that glibenclamide, but not nateglinide, induced β-cell apoptosis in cultured human islets [5]. In Japan, approximately half of all patients with diabetes have a genetic predisposition to the disease, and insulin secretion is often impaired in lean patients with diabetes mellitus [6,7]. Additionally, Japanese and Asian patients often show reduced β cell function. Mitiglinide is a short-acting insulinotropic agent used in type 2 diabetes treatment. It has a rapid stimulatory effect on insulin secretion and reduces postprandial plasma glucose level in patients with type 2 diabetes [8].

Based on these results, we hypothesized that glinide may be superior to sulfonylurea about beta-cell dysfunction in Japanese patients with type 2 diabetes mellitus. Therefore, we evaluated the metabolic effect of glinide/sulfonylurea (SU) using the Japan Diabetes Society-developed meal tolerance test (MTT) in Japanese patients with type 2 diabetes mellitus, which shows a tight correlation with glucose tolerance test.

## Methods

### Subjects

Twenty-two outpatients with type 2 diabetes mellitus participated in this study at Tottori University Hospital. Type 2 diabetes mellitus was diagnosed based on the criteria of the Japan Diabetes Society [9]. These patients were divided into mitiglinide group and glimepiride group. The patients were continuously assigned to the mitiglinide group and the glimepiride group. First 15 patients were assigned to the mitiglinide group and 7 patients who were registered thereafter were assigned to the glimepiride group. These patients were given mitiglinide 30 mg/day, or glimepiride 0.5 mg/day. This study was a prospective, open label, non-randomized, clinical study. There were fifteen patients in mitiglinide group and seven patients in glimepiride group. There were ten males and five females in mitiglinide group, and six males and one female in glimepiride group. The mean age of the patients in mitiglinide group was 58.5 years, mean BMI was 24.8 kg/m^2^, mean fasting plasma glucose was 6.88 mmol/L (124 mg/dl), mean HbA1c was 6.80% (50.8 mmol/mol) (Table 1). The mean age of the patients in glimepiride group was 65.1 years, mean BMI was 26.3 kg/m^2^, mean fasting plasma glucose was 8.16 mmol/L (147 mg/dl), mean HbA1c was 7.60% (59.5 mmol/mol) (Table 1). Patients with pancreatic disease, liver disease, renal failure, or those taking diabetogenic medications such as corticosteroids were excluded from this study. All patients were diabetic drug naïve patients.

**Table 1:** Patient characteristics

	**Mitiglinide**	**Glimepiride**
n (M/F)	15 (10/5)	7 (6/1)
Age, years	58.5 ± 11.5	65.1 ± 7.5
BMI, kg/m^2^	24.8 ± 4.0	26.3 ± 6.2
SBP, mmHg	137 ± 17	132 ± 20
DBP, mmHg	80 ± 7	79 ± 11
Total cholesterol, mmol/L	5.38 ± 0.98	5.43 ± 1.08
(mg/dL)	(208 ± 38)	(210 ± 42)
Triglycerides, mmol/L	1.71 ± 1.14	1.89 ± 0.74
(mg/dL)	(152 ± 101)	(168 ± 66)
HDL-C, mmol/L	1.39 ± 0.38	1.37 ± 0.31
(mg/dL)	(54 ± 15)	(53 ± 12)
Fasting glucose, mmol/L	6.88 ± 1.27	8.16 ± 1.05
(mg/dL)	(124 ± 23)	(147 ± 19)
2-h postload glucose, mmol/L	9.11 ± 1.94	11.6 ± 1.77
(mg/dL)	(164 ± 35)	(209 ± 32)
HbA1c(NGSP),%	6.80 ± 0.54	7.60 ± 0.63
Fasting IRI, pmol/L	52.7 ± 53.4	49.3 ± 36.1
(ng/mL)	(7.6 ± 7.7)	(7.1 ± 5.2)
HOMA-R	2.55 ± 3.10	2.67 ± 2.09
ISI (Matsuda’s index)	7.73 ± 3.16	7.68 ± 4.69
Insulinogenic index	0.80 ± 0.61	0.42 ± 0.38
CPR, nmol/L	0.59 ± 0.30	0.57 ± 0.17
(ng/mL)	(1.79 ± 0.93)	(1.72 ± 0.51)
Proinsulin, pmol/L	4.29 ± 4.16	4.77 ± 4.04
PI/I ratio	0.10 ± 0.11	0.19 ± 0.26

Data are means ± standard deviation.

BMI, body mass index; SBP, systolic blood pressure; DBP, diastolic blood pressure; HDL-C, high-density lipoprotein cholesterol; HbA1c, hemoglobin A1c; NGSP, National Glycohemoglobin Standardization Program; IRI, immunoreactive insulin; HOMA-IR, homeostasis model assessment of insulin resistance; ISI, insulin sensitivity index; CPR, C-peptide immunoreactivity; PI/I ratio, proinsulin/insulin ratio.

This study was approved by the Ethics Committee of the Faculty of Medicine, Tottori University. Informed consent was obtained from all of the patients using a procedure approved by the Ethics Committee. This study was registered in University Hospital Medical Information Network Clinical Trials Registry (UMIN-CTR), the ID was UMIN000010467.

### Meal tolerance test

After fasting for at least 12 h, the participants visited the clinic in the morning and consumed a test meal prepared by the Japan Diabetes Society (450 kcal/1882 kJ; 15% protein, 35% fat, and 50% carbohydrate; 1.6 g salt x1.6 g) [10]. Plasma glucose and insulin were measured at 0 (fasting), 30, 60, 120, and 180 min after the test meal. Serum C-peptide immunoreactivity (CPR) was measured at 0 (fasting) and 120 min. Plasma glucose was measured using the glucose oxidase method. Plasma insulin and CPR levels were measured using chemiluminescent immunoassays (CLIA) (human insulin and CPR CLIA kits; Kyowa Medix, Tokyo, Japan). Plasma insulin was defined as immunoreactive insulin (IRI). Plasma proinsulin was measured using an enzyme-linked immunosorbent assay (ELISA) (human intact proinsulin ELISA kit; Biovendor Heidelberg, Germany). This method of meal tolerance test (MTT) was a well-established method in our hospital as previously reported [11].

HbA1c (JDS: Japan Diabetes Society) was measured by high-performance liquid chromatography and was converted to National Glycohemoglobin Standardization Program (NGSP) values using the following officially certified equation: NGSP (%) = 1.02 × JDS (%) + 0.25% [12]. The reverse equation is: JDS (%) = 0.980 × NGSP (%) − 0.245%.

### Calculation of insulin secretion and resistance indices

Insulinogenic Index = [(insulin at 30 min) - (insulin at 0 min)]/[(glucose at 30 min) - (glucose at 0 min)] [13].

HOMA-IR = [fasting plasma glucose (FPG; mmol/L)] × [fasting IRI (F-IRI; pmol/L)]/135 [14].

ISI = 10,000/√{[FPG (mmol/L) × FPI (pmol/L)] × [mean glucose × mean insulin during the MTT]} [15].

### Self-monitoring blood glucose (SMBG)

SMBG was performed at 7 times/day (before and after every meal, bedtime). Patients checked their blood glucose level with a SMBG device (Freestyle, Nipro, Osaka, Japan). Mean blood glucose of 3 days was calculated at 7 points.

### Statistical analysis

Data are expressed as means ± standard error of the mean. Two groups were compared using paired Student’s t-test. Values of *P* < 0.05 were considered significant. SPSS software version 15.0 (SPSS, Chicago, IL, USA) was used for all analyses.

## Results

### Meal tolerance test

**Figure 1:**
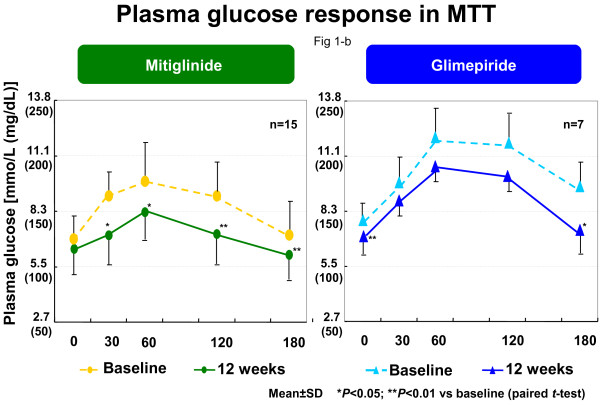
**Plasma glucose response in meal tolerance test.** Plasma glucose level at 60, 120, and 180 min was significantly improved by mitiglinide **(a)**. Whereas, glimepiride showed a significant improve plasma glucose at only 180 min **(b)**.

**Figure 2:**
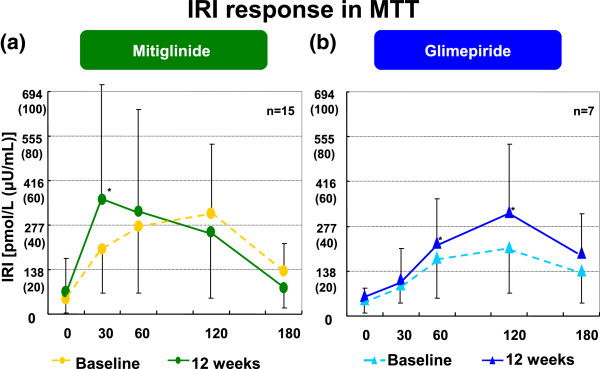
**IRI response in meal tolerance test.** Peak IRI shifted from 120 to 30 min by mitiglinide treatment **(a)**. The pattern of insulin secretion was not changed by glimepiride treatment **(b)**.

Plasma glucose level at 30, 60, 120, and 180 min was significantly improved by mitiglinide (Figure 1-a). Whereas, glimepiride showed a significant improve plasma glucose at 0, 180 min (Figure 1-b). Peak IRI shifted from 120 to 30 min by mitiglinide treatment, the insulin level in 30 min significantly increased (Figure 2-a). The pattern of insulin secretion was not changed by glimepiride treatment, and the IRI level significantly increased at 60, 120 min (Figure 2-b).

### Self-monitoring blood glucose

**Figure 3:**
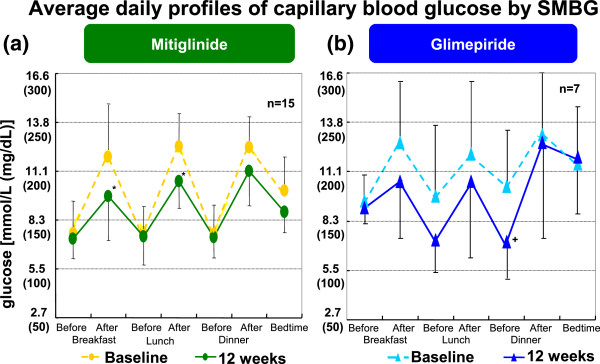
**Average daily profiles of capillary blood glucose by SMBG.** Postprandial glucose level was significantly improved by mitiglinide at all mealtimes **(a)**. In contrast, glimepiride reduced both pre- and post-prandial glucose level, and there was significant reduction of blood glucose level in before dinner time **(b)**.

**Figure 4:**
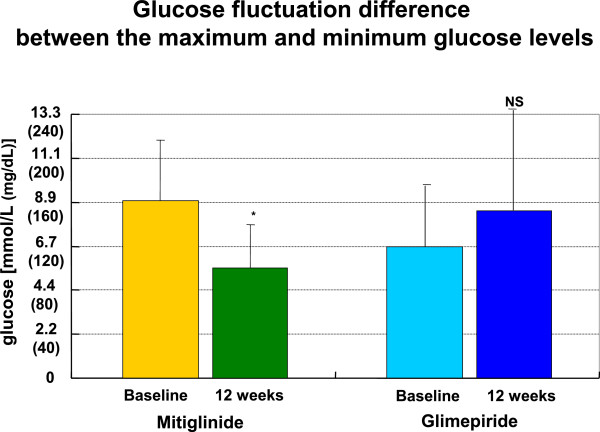
**Glucose fluctuations difference between maximum and minimum glucose levels in SMBG.** Mitiglinide but not glimepiride improved postprandial glucose level by SMBG.

Postprandial glucose level was significantly improved by mitiglinide at all mealtimes (Figure 3-a). In contrast, glimepiride reduced both pre- and post-prandial glucose level, and there was significant reduction of blood glucose level only in before dinner time (Figure 3-b). Mitiglinide but not glimepiride improved glucose fluctuations difference between maximum and minimum glucose levels (Figure 4-a,b).

### Proinsulin/Insulin ratio

**Figure 5:**
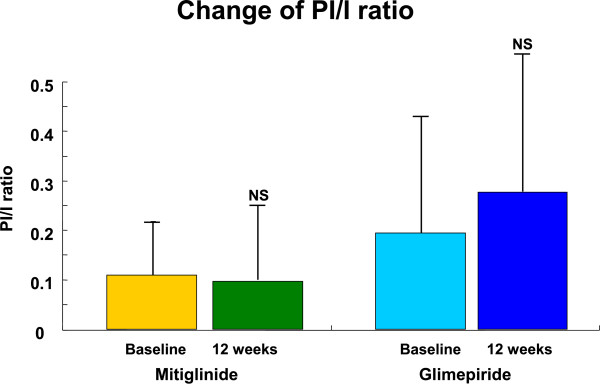
**Change of PI/I ratio.** Whereas mitiglinide did not affect the PI/I ratio, glimepiride tended to increase this parameter.

**Figure 6:**
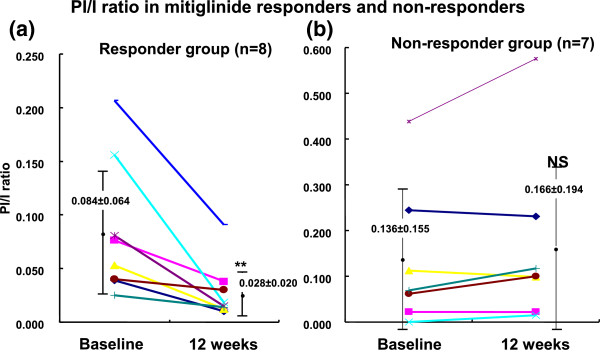
**PI/I ratio in mitiglinide responders (non-responders).** PI/I ratio was reduced significantly in the responder group **(a)**. PI/I ratio did not change in the non-responder group **(b)**.

Whereas mitiglinide did not affect the PI/I ratio, glimepiride tended to increase PI/I ratio (Figure 5). Moreover, although mitiglinide did not affect PI/I ratio as a whole, marked reduction was noted in some patients treated by this agent (Figure 6). PI/I ratio was reduced significantly in the responder group (0.084 +/− 0.064 to 0.028 +/− 0.020, P < 0.01). (Responders defined as patients in whom PI/I ratio was reduced >10% vs baseline by mitiglinide treatment.)

The responder subgroup exhibited significantly higher insulin sensitivity index (9.30 +/− 2.35 vs 6.15 +/− 3.23, P < 0.05) and higher insulinogenic index (1.01 +/− 0.69 vs 0.43 +/− 0.30, P < 0.05) at baseline than non-responders (Table 2). The triglyceride (TG) level of responders was significantly lower than that of non-responders (1.19 ± 0.50 vs 2.32 ± 1.40, P < 0.05).

**Table 2:** Comparison of baseline metabolic profile between P/I ratio responders and non-responders

	**Responder**	**Non responder**
n (M/F)	8 (5/3)	7 (5/2)
BMI, kg/m^2^	23.6 ± 4.2	26.4 ± 4.1
HbA1c(NGSP),%	6.8 ± 0.4	6.9 ± 0.7
HOMA-R	1.85 ± 1.18	3.90 ± 4.58
ISI (Matsuda’s index)	9.30 ± 2.35	6.15 ± 3.23*
Insulinogenic index	1.01 ± 0.69	0.43 ± 0.30*
PI/I ratio	0.08 ± 0.06	0.13 ± 0.15
CPR, nmol/L	0.66 ± 0.33	0.66 ± 0.33
(ng/dL)	(2.0 ± 1.0)	(2.0 ± 1.0)
Total cholesterol, mmol/L	5.18 ± 1.21	5.54 ± 0.59
(mg/dL)	(200 ± 47)	(214 ± 23)
Triglyceride, mmol/L	1.19 ± 0.50	2.32 ± 1.40*
(mg/dL)	(106 ± 45)	(206 ± 124*)
HDL-C, mmol/L	1.73 ± 0.51	1.16 ± 0.31*
(mg/dL)	(67 ± 20)	(45 ± 12*)

Mean ± SD *P < 0.05 vs responder (paired *t*-test).

BMI, body mass index; HbA1c, hemoglobin A1c; NGSP, National Glycohemoglobin Standardization Program; HOMA-IR, homeostasis model assessment of insulin resistance; ISI, insulin sensitivity index; PI/I ratio, proinsulin/insulin ratio; CPR, C-peptide immunoreactivity; HDL-C, high-density lipoprotein cholesterol; IRI, immunoreactive insulin.

## Discussion

In this study, postprandial glucose level was significantly reduced by mitiglinide treatment. After 3 months treatment, mitiglinide did not increase PI/I ratio. In contrast, glimepiride increased PI/I ratio. Interestingly, there were two distinct subgroups among patients treated by mitiglinide about the PI/I ratio: responders and non-responders. In the responders subgroup, marked reduction of PI/I ratio was observed. The responder subgroup exhibited less insulin resistance and the higher insulinogenic index at baseline than non-responders. Moreover, the TG level of responders was significantly lower than that of non-responders.

An elevated PI/I ratio reflects impairment of insulin secretory capacity of β-cells and cardiovascular risk in patients with IGT or T2DM. Mitiglinide stimulates β-cells and quickly accelerates insulin secretion, which is the physiological property of these cells. Kawai et al. reported that treatment of GK rats with nateglinide and glibenclamide varies in long-term effects on β-cell functions; nateglinide treatment better preserved pancreatic islet morphology than did glibenclamide treatment [16]. Laghmich et al. reported that when both normal and GK rats were exposed to nateglinide and glibenclamide for 7 days, insulin in islets, the secretory response to high-glucose and basal biosynthetic activity were better in those animals which received nateglinide [17]. Taken together, these data suggest that short-acting nateglinide preserves β-cell functions better than the long-acting glibenclamide does. RØDER et al. reported that the fasting PI/IRI ratio appears to be a marker of the degree of reduced maximal acute insulin response (AIR max) in type 2 DM [18]. Mitiglinide reduces the levels of circulating biomarkers of oxidative stress and inflammation caused by postprandial hyperglycemia [19]. We speculate that mitiglinide might rescue impairment of β-cell function possibly via preventing postprandial stress.

In this study, the insulin resistance level of responders was significantly lower than that of non-responders. Haffner and colleagues examined the relation between the fasting proinsulin-to-insulin ratio with a number of metabolic disorders believed to be associated with the insulin resistance (IR) syndrome. In 423 subjects without diabetes, an increased ratio was significantly associated with hypertension, low high-density lipoprotein cholesterol, high triglyceride levels, and impaired glucose tolerance. These results suggest that even nondiabetic individuals with the IR syndrome not only exhibit hyperinsulinemia as a marker of IR, but also show elevated proinsulin values, which may reflect relative beta-cell failure or malfunction. [20]. Another investigation explored the predictive value of intact proinsulin in 48 T2DM patients. There was a significant correlation between intact proinsulin values and insulin resistance measurement of minimal model analysis [21]. These results might be able to explain the PI/I ratio non-responder group showed higher insulin resistance.

Next, one study reported that metformin treatment in subjects with Type 2 diabetes mellitus significantly reduced concentrations of proinsulin-like molecules over a 12-week period. They conclude that short-term effects of metformin treatment on proinsulin-like molecules are similar to those previously observed with dietary treatment in subjects with Type 2 diabetes but opposite to those of sulphonylurea treatment. [22]. Furthermore, several studies reported that treatment with pioglitazone resulted in significant decreases in elevated proinsulin levels in type 2 diabetes patients. This effect was independent from glycemic control [23]. These results suggest that the treatment of insulin resistance is important for reduction of PI/I ratio.

In this study, the TG level of responders was significantly lower than that of non-responders. Gama et al. reported that fasting intact proinsulin concentrations were similar in hypertriglyceridaemic subjects with normal glucose tolerance and control subjects but these were lower than in hypertriglyceridaemic subjects with impaired glucose tolerance [24]. Moreover, although hyperproinsulinaemia has been reported in hypertriglyceridaemic subjects with type 2 diabetes mellitus, this is probably related to their glucose intolerance rather than their hypertriglyceridemia [25]. These results suggest that poor controlled type 2 DM with hypertriglycemia induce hyperproinsulinaemia. These results might be able to explain that the TG level of responders was lower than that of non-responders in patients with type 2 DM in this study,

RØDER et al. reported that the PI/IRI ratio correlated inversely with the acute insulin response (AIR) max in the T2DM patients. They conclude that the magnitude of the elevation in fasting PI/IRI is related to the reduction in AIR max. Thus, the fasting PI/IRI ratio appears to be a marker of the degree of reduced AIR Max in T2DM [18]. These results might be able to explain the PI/I ratio non-responder group showed a lower insulinogenic index. It has been proposed that the restoration of both the early phase of insulin release and postprandial hyperglycemia have potentially significant implications in improving metabolic control and reducing macrovascular complications [26]. Current evidence suggests that proinsulin contributes to the excess incidence of cardiovascular disease in T2DM by stimulating plasminogen activator inhibitor-1 secretion and the consecutive inhibition of fibrinolysis [27]. Our results suggest that mitiglinide treatment contribute to improve metabolic control and cardiovascular risk reduction in patients with type 2 diabetes mellitus.

Our study had several limitations, including the small number of patients and short term study. The major limitation of our study is the small number of patients. As only 15 patients participated glinide group in this study, and PI/I responder subgroup was only 8 patients, and the glimepride group was only 7 patients. Our study may have biases, because of the small number of patients. Our results require confirmation in a larger study. If larger number of patients was observed, the PI/I ratio of SU group might significantly increase. Furthermore, the background between the mitiglinide group and the glimepiride group was different. Randomized controlled study or cross-over study is desired.

Because the term of this study was only 3 months, our study needs longer term to confirm our results. If longer term was observed, the PI/I ratio of SU group might significantly increase. Despite these limitations, we think that our study may aid routine clinical treatment of Japanese and other Asian patients with type 2 diabetes mellitus.

## Conclusion

Mitiglinide improved postprandial insulin secretion pattern and thereby suppressed postprandial glucose spike. In T2DM patients with low insulin resistance and low TG, mitiglinide recovered impaired β-cell function from the viewpoint of the PI/I ratio.

## Abbreviations

BMI: Body mass index; CPR: C-peptide immunoreactivity; FPG: Fasting plasma glucose; HDL-C: High-density lipoprotein cholesterol; HOMA-IR: Homeostasis model assessment for insulin resistance; IRI: Immunoreactive insulin; ISI: Insulin sensitivity index; MTT: Meal tolerance test; PPG: Postprandial plasma glucose; TG: Triglyceride.

## Competing interests

The authors declare that they have no competing interests.

## Authors’ contributions

TO and KI participated in the design of the study and performed the statistical analysis. YF, HS, KS, NY, KM, SI, and HO collected the data. MK, KY, and ST conceived the study, participated in its design and coordination, and helped to draft the manuscript. All authors read and approved the final manuscript.

## Author details

Tsuyoshi Ohkura, Assistant Professor, Division of Cardiovascular Medicine, Endocrinology and Metabolism, Department of Molecular Medicine and Therapeutics, Tottori University Faculty of Medicine, Nishi-chou 36–1, Yonago, Tottori 683–8504, Japan.

Tel: +81-859-38-6517; Fax: +81-859-38-6519.
